# Targeting MYCN and ALK in resistant and relapsing neuroblastoma

**DOI:** 10.20517/cdr.2019.009

**Published:** 2019-09-19

**Authors:** Elizabeth R Tucker, Evon Poon, Louis Chesler

**Affiliations:** Division of Clinical Studies, The Institute of Cancer Research, Sutton, SM2 5NG, UK.

**Keywords:** Neuroblastoma, anaplastic lymphoma kinase, *MYCN*, therapeutics

## Abstract

Neuroblastoma, a tumor of peripheral nerve, is the most common solid tumor of young children. In high-risk disease, which comprises approximately half of patients, death from chemotherapy-resistant, metastatic relapse is very frequent. Children who relapse exhibit clonal enrichment of two genomic alterations: high-level amplification of the *MYCN* oncogene, and kinase domain mutations of the anaplastic lymphoma kinase (*ALK*) gene. Overall survival in this patient cohort is less than 15% at 3 years, and there are few options for rationally targeted therapy. Neuroblastoma patients exhibit *de novo* resistance to many existing *ALK* inhibitors, and no clinical therapeutics to target *MYCN* have yet been developed. This review outlines the international efforts to uncover mechanisms of oncogenic action that are therapeutically targetable using small-molecule inhibitors. We describe a mechanistic interaction in which *ALK* upregulates *MYCN* transcription, and discuss clinical trials emerging to develop transcriptional inhibitors of *MYCN*, and to identify effective inhibitors of *ALK* in neuroblastoma patients.

## Introduction

Neuroblastoma is a malignancy of the developing sympathetic nervous system with up to 100 UK children newly diagnosed each year. The risk stratification of neuroblastoma is highly complex and under constant review in order to identify children who may require more aggressive treatment up-front in an effort to prevent relapse, which is almost uniformly fatal. Most recently, a subgroup of ultra-high-risk patients was proposed, based upon age greater than 5 years, high serum lactate dehydrogenase and the involvement of at least one metastatic site, to enable clinicians to pick out these patients at diagnosis for early referral to novel therapeutic trials^[1]^. However, there is also increasingly robust pre-clinical and clinical evidence of molecular subgroups which predict poor outcome, not least the ultra-high-risk patient cohort exhibiting both amplification of *MYCN* and kinase domain mutations of anaplastic lymphoma kinase (*ALK*)^[2,3]^. Herein we discuss the consequences of *MYCN* amplification and expression of the *ALK* tyrosine kinase both individually and concomitantly, and highlight the small molecule strategies under investigation to target these aberrations.

## Novel therapeutic approaches for *MYCN*-amplified neuroblastoma

Amplification of *MYCN* is a defining feature of high-risk neuroblastoma, which when present at diagnosis, predicts a five-year overall survival of only 50%^[4]^. Tumors with amplification of *MYCN* are also more likely to exhibit unfavorable histology, diploidy, 1p deletion and 17q gain, all of which are associated with poor prognosis. Multi-modality high-risk treatment regimens for these children are contributing to improved outcomes, but it is hoped that clinical implementation of novel targeted therapeutics will have a greater impact for this group of patients. Various strategies to therapeutically down-regulate the activity of *MYCN* have been the subject of multiple preclinical studies, but few of these have progressed to tangible clinical trials^[5]^. The development of MYCN-targeted drugs has been hindered by the complexity and variability of primary MYC structure in solution. As a result, despite being an attractive therapeutic target, there are no clinically available drugs that directly target MYCN. However, many promising approaches to target *MYCN* indirectly and its transcriptional output have been developed. These mainly act by blocking *MYCN* stability, using transcriptional inhibitors or targeting synthetic lethal interactions.

The *MYC*-family of oncoproteins are stabilized by altered phosphorylation at the conserved T58 and S62 residues^[6]^. Signaling via the PI3K/Akt pathway in neuroblastoma regulates the phosphorylation of MYCN through GSK3b and mTOR, which makes this pathway a suitable candidate for pharmacological inhibition in order to indirectly target MYCN stability. Preferential sensitivity to inhibitors of PI3K/mTOR, including NVP-BEZ235, were prominently identified in a chemical-genetic screen of isogenic neuroblastoma cells with genetically modified MYCN stabilization versus wild-type MYCN expression^[7]^. NVP-BEZ235 went on to demonstrate growth inhibition of neuroblastoma cells via suppression of MYCN both *in vitro* and *in vivo*. Whilst NVP-BEZ235 is not a clinical candidate compound, the PI3K/mTOR inhibitor SF1126, was taken into pediatric trials for relapsed or refractory neuroblastoma (NCT02337309) (see Table 1 for full summary of targeted inhibitors in clinical studies for neuroblastoma). Recruitment to this study did not meet expectations, but it was the first pediatric study to feature the biomarkers PI3K/AKT/mTOR which can be measured from disseminated tumor cells or platelet-rich plasma^[8]^. The more potent TORC1/TORC2 inhibitor, AZD2014, has also been added to the European Proof-of-Concept therapeutic Stratification Trial of Molecular Anomalies in Relapsed or Refractory Tumors (ESMART, NCT02813135). This trial aims to provide targeted therapy options for pediatric patients with molecular anomalies in their tumors which are not actionable via any other open study for children in Europe.

**Table 1:** Summary of open and completed trials for *MYCN* or *ALK*-activated neuroblastomas

Compound	Pediatric Phase	Identifier	Target	Summary of outcome	Notes
**Indirect therapeutic targeting of MYCN**					
SF1126	I	NCT02337309	PI3K/mTOR	Terminated	
AZD2014	I/II	NCT02813135	TORC1/TORC2	Recruiting	ESMART: basket trial to cover the targeting of different survival pathways in pediatric cancers
Alisertib	II	NCT02444884; NCT01154816; NCT01601535; NCT01601535	Aurora A Kinase	Completed	Poorly tolerated, with activity not linked to *MYCN* status.
AT9283	I	EudraCT2008-005542-23	Aurora A/B Kinase	Completed	Target inhibition demonstrated by reduction in phosphor-histone 3 in paired skin punch biopsies.
GSK525762	I	NCT01587703	BET	Completed	16 years and above
LY3023414	II	NCT03155620	PI3K/mTOR	Recruiting	LY3023414 is one in a panel of compounds being investigated in The Pediatric MATCH Screening Trial, for refractory or relapsing pediatric cancers
**Therapeutic targeting of ALK**					
Crizotinib	I	NCT01606878	*ALK*	Completed	Crizotinib and combination chemotherapy in younger patients with relapsed or refractory solid tumors or Anaplastic Large Cell Lymphoma
Crizotinib (in combination with temsirolimus)	Ib	ITCC053	*ALK*/ROS1/MET	Recruiting	For relapsed/refractory neuroblastoma or rhabdomyosarcoma
Crizotinib	II	NCT02559778	*ALK*	Recruiting	PEDS-PLAN: evaluating feasibility of molecularly guided therapy in combination with induction chemotherapy
Crizotinib	III	NCT03126916	No molecular subgroup defined	Recruiting	Testing Crizotinib or Iobenguane I-131 with standard therapy for patients with high-risk neuroblastoma
Ceritinib	I	NCT01742286	*ALK*	Recruiting	For pediatric patients with ALK-activated tumors
Ceritinib (in combination with Ribociclib)	I	NCT02780128	*ALK* & CDK4/6	Recruiting	NEPENTHE: to match genomic aberrations at time of relapse to rationally designed combinations
Lorlatinib	I	NCT03107988	*ALK*	Recruiting	Lorlatinib as a single agent and in combination with chemotherapy in children with relapsed/refractory neuroblastoma
Entrectinib	I	NCT02650401	*ALK* fusions, TRK & ROS1	Recruiting	For recurrent or refractory solid tumors, with or without TRK, ROS1 or ALK fusions
Ensartinib, Selumetinib sulfate & Ulixertinib	II	NCT03155620	*ALK*, activating MAPK pathway mutations and MAPK pathway mutations respectively	Recruiting	Ensartinib, Selumetinib sulfate and Ulixertinib are in a larger panel of compounds being investigated in The Pediatric MATCH Screening Trial, for refractory or relapsing pediatric cancers

The control of MYCN expression has been further clarified in recent years. During the cell cycle MYCN stability is directly controlled by Aurora-A which competes with the E3 ligase FBXW7 to prevent the proteosomal degradation of MYCN^[9-12]^. Interestingly, Aurora-A is expressed at an elevated level in *MYCN*-amplified neuroblastoma^[12]^. We and others have reported that allosteric Aurora-A inhibitors (such as Alisertib and CD532) can dissociate the interaction between Aurora-A and MYCN, resulting in degradation of MYCN and reduced transcriptional output of MYCN^[10,11]^. Following this, two Aurora inhibitors have been clinically evaluated in pediatrics; AT9283 (EudraCT2008-005542-23), an Aurora-A/B inhibitor and Alisertib (NCT02444884, NCT01154816, NCT01601535, NCT01601535), a specific Aurora-A inhibitor. However both compounds were not well tolerated in pediatric Phase I and II trials^[13-17]^. MYCN status did not improve response and it is unknown whether MYCN is selectively targeted in these tumors. The way remains open for suggestions of alternative or combination studies. For example, it is predicted that the combination targeting of Aurora-A and ATR is beneficial in MYCN-driven tumors, as Aurora-A inhibitors activates ATR^[9]^.

Recently, a set of self-regulated master transcription factors known as the “core transcriptional regulatory circuits” have been described to maintain *MYCN*-amplified neuroblastoma in a state of pro-growth and pro-survival^[18]^. The identification of the key genes involved in maintaining these tumors will allow researchers to focus on finding specific vulnerabilities in the pathways that they control. In addition, indirect approaches to block transcriptional output of MYCN have gained traction with the development of improved genetic and chemical tools targeting various components of the transcriptional machinery that chaperone MYC to target promotors and enhancers.

Inhibition of the bromodomain and extraterminal domain (BET) family has also been shown to down-regulate *MYCN* transcription in *MYCN*-amplified neuroblastoma^[19]^. Bromodomain-4 (BRD4), a member of this family, interacts with the positive transcription elongation factor b, and together they are recruited to promoters to phosphorylate RNA polymerase II, particularly at the promoters of genes associated with super-enhancer regions, including MYC^[20,21]^. In an effort to support the progression of BRD4 inhibitors to the pediatric clinic for neuroblastoma, OTX015, an orally-administered compound, was studied in a panel of preclinical neuroblastoma models^[22]^. This molecule was found to have specific activity against MYCN target genes, which correlated with high level MYCN expression and *MYCN* amplification in a panel of neuroblastoma cell lines. The conclusions from a recent phase I/II study with the BET inhibitor, GSK525762, which allowed for the inclusion of neuroblastoma patients over the age of 16, are awaited (NCT01587703).

Cyclin dependent kinase 7 (CDK7) and cyclin dependent kinase 9 (CDK9) play a key role in the transcriptional cycle of RNA polymerase II. Inhibition of CDK7 or CDK9 selectively kills tumor cells, by targeting the super-enhancer clusters of the genome that are associated with MYCN regulation^[23]^. In the laboratory, many mono-or pan-CDK inhibitors have been reported to display robust anti-tumor effects either by down-regulating MYCN protein or shutting down its transcriptional activity in neuroblastoma (THZ1, CYC065 and dinaciclib) or other cancers (SY-1365, BAY1143572)^[24]^. Although many of them are undergoing clinical evaluation in adults, pediatric trials in neuroblastoma have not yet commenced.

Genetic screens have been used to identify genes that are synthetic lethal to *MYCN* amplification/overexpression, leading to preclinical evaluation of many new agents in neuroblastoma. One of such agent is CCT244747 following the characterization of synthetic lethal interaction between CHK1 and MYCN^[25]^. Another example is AT7519 following the finding that inactivation of CDK2 is synthetically lethal to *MYCN* in neuroblastoma cells^[26]^. These new approaches may identify many new targets and agents that are effective in treating neuroblastoma, especially when used in combination with established therapeutics^[27]^.

*MYCN* amplification and overexpression has also been found in a further small subset of pediatric and adult cancers such as medulloblastoma, retinoblastoma, glioma, lung, pancreas, prostate and hematological cancers. Some effort has been made in the recent years to inhibit MYCN in these tumors, however, most of them were adapted from approaches that have been identified in research conducted originally in neuroblastoma^[28]^.

## Direct and combinatorial therapeutics for *ALK*-mutant neuroblastoma

**Figure 1:**
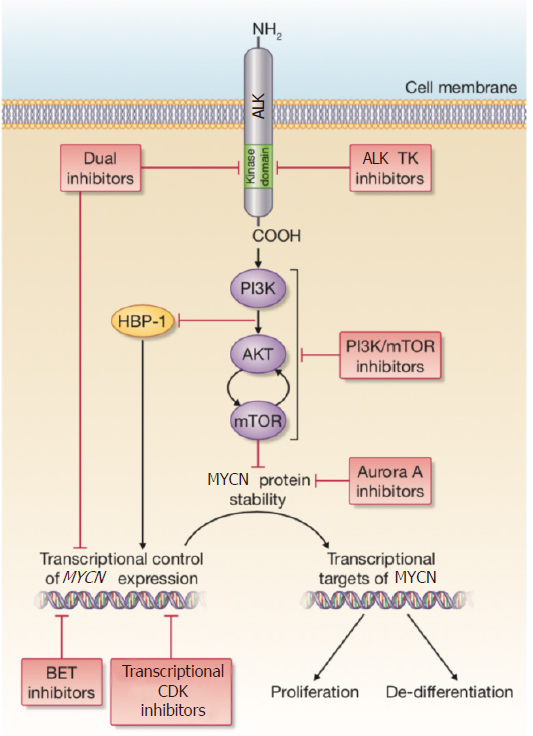
A summary of the signaling connections between *ALK* and *MYCN* in neuroblastoma. The red boxes describe current therapeutic approaches that are being pursued either preclinically or in clinical studies

Mutations of the tyrosine kinase ALK were firstly identified in neuroblastoma as the main cause of rare, familial cases of the disease^[29,30]^. In hereditary neuroblastoma, a mutation at the R1275 locus of *ALK* can be treated using the first-generation ALK inhibitor, Crizotinib^[31]^. In non-hereditary cases of neuroblastoma somatic *ALK* mutations or amplifications have been shown to be associated with a poor prognosis, for which Crizotinib treatment is often not efficacious^[2,30,32,33]^. *ALK* mutations are found in around 9% of all neuroblastomas at diagnosis, with the incidence increasing to 14% in the high-risk subtype^[2]^. In contrast to the situation with *MYCN* amplification, ALK is the first kinase identified as a driver in neuroblastoma with real potential as a tractable therapeutic target, due to the number of inhibitors already available. If *MYCN* amplification and an *ALK* mutation are found concomitantly, this is associated with an ultra-high-risk molecular phenotype^[3]^. Constitutive activity of ALK, which activates signaling via PI3K/Akt, MAPK, ERK5 and JAK/STAT, leads to the transcriptional up-regulation of *MYCN* and MYCN protein stabilization, thus compounding the aggressive nature of this cohort of tumors^[34-36]^ [Figure 1].

The availability of later-generation ALK-targeted therapeutics has led to a unique situation for pediatric neuroblastoma patients. There are now open trials for pediatric patients with either Ceritinib or Lorlatinib. Ceritinib, which received USA Food and Drug Administration approval in 2014 for treatment of patients with *ALK*-rearranged metastatic non-small cell lung cancer (NSCLC), has shown efficacy above that of Crizotinib in neuroblastoma models^[37]^. However, its success in neuroblastoma patients is yet not fully evaluated, apart from a favorable case report for a tumor harboring an *ALK* I1171T mutation^[38]^. I1171T is a gain-of function *ALK* mutation, located in the neuroblastoma mutation hotspot of ALK, at the αC-helix in the amino-terminal lobe of the kinase. The most common neuroblastoma-associated mutations at this hotspot are F1174, R1275 and F1274, each with different degrees of transforming ability and differential sensitivities to available inhibitors^[2,39]^. A screen of known neuroblastoma-associated *ALK* mutations utilizing recombinant ALK variants, revealed that response to crizotinib correlated with a relatively reduced affinity of the mutant kinase for ATP. For example, *ALK* F1174L results in higher ATP-binding affinity, predicting resistance to crizotinib^[2]^. Much attention has been upon the potential of the third-generation ALK inhibitor, Lorlatinib, for which exceptional preclinical evidence of its efficacy against the Crizotinib-resistant ALK F1174L has been published^[40,41]^. Lorlatinib was initially developed for NSCLC patients requiring targeted therapy against ALK for brain tumor metastases, as it has good Central nervous system (CNS) availability^[42]^. This could become a necessity for children with neuroblastoma, as relapses within the CNS may become more frequent following the introduction of immunotherapy into standard therapy. Based on this evidence, Lorlatinib is now available in an international study for children with relapsed *ALK* mutant neuroblastoma. Other ALK inhibitors demonstrating good preclinical rationale for neuroblastoma include Alectinib and Entrectinib^[43,44]^. Alectinib shows activity against RET, a downstream target of ALK in neuroblastoma, which may increase its effectiveness in this form of the disease^[45]^. Entrectinib inhibits both ALK and the TRK receptors; TRKb is associated with poor prognosis in neuroblastoma. Both these compounds are therefore a priority for further pediatric studies.

Although the standard route for the evaluation of novel compounds is through early phase clinical studies in relapsing or treatment-refractory disease, there is strong preclinical rationale for the combination of ALK inhibition with up-front chemotherapy in *ALK*-positive neuroblastoma patients. Krytska *et al*.^[46]^ demonstrated that neuroblastoma *ALK* cell lines with *de novo* resistance to Crizotinib, achieved complete responses to Crizotinib combined with chemotherapy in xenograft models. This synergy was dependent upon a functional p53 pathway, further making the case for targeted therapy upfront in treatment schedules, as p53 pathway in-activation is a characteristic of chemotherapy-resistant disease^[47]^. The phase II pediatric study, PEDS-PLAN, is currently evaluating feasibility of molecularly guided therapy in combination with induction chemotherapy (NCT02559778). Additionally, Crizotinib is being tested against Iobenguane I-131 with standard therapy for children newly diagnosed with high-risk neuroblastoma or ganglioneuroblastoma (NCT03126916).

In *ALK*-rearranged NSCLC, there is a strong precedent for the transition of patients between different ALK inhibitors, as a common mechanism of resistance in this patient group is the acquisition of a secondary treatment-induced *ALK* mutation^[48]^. It has even been reported that re-challenge with a previously used ALK inhibitor can be effective, if compound *ALK* mutations occur with different inhibitor sensitivities^[49]^. In neuroblastoma, the picture of acquired resistance to ALK inhibitors may be very different. Upregulation of signaling through the alternative tyrosine kinase AXL, associated with an increase in endothelial-to-mesenchymal transition, has been proposed in response to induced resistance to the non-clinical ALK inhibitor TAE684, *in vitro*^[50]^. A more recent study of induced Lorlatinib resistance *in vitro* pin-pointed an acquired mutation of *NF1* with RAS/MAPK activation^[51]^.

In fact, the dominance of the RAS/MAPK pathway in refractory and relapsing neuroblastoma is becoming clear. Relapsed high-risk neuroblastoma has a higher mutational burden than neuroblastomas at diagnosis, and the largest study of this type has shown that over half of the mutations identified at relapse are targetable by compounds already in clinical development^[52]^. This study also concurred with evidence of the preponderance of RAS/MAPK pathway mutations at relapse published previously^[53-55]^. For example, Eleveld *et al*.^[54]^ carried out a whole genome sequencing paired study of 23 diagnostic and relapsing neuroblastomas, finding that 18 out of 23 relapse samples had acquired mutations predicted to hyperactivate the RAS/MAPK pathway. Included in this relapse group were 10 mutations of *ALK*, strongly suggesting that the incidence of *ALK* mutations at relapse is higher than at diagnosis. Re-sequencing of tumors in relapsing patients is therefore essential, due to the availability of ALK inhibitors, amongst others. However, the application of MEK inhibitors to treat *ALK*-activated neuroblastomas is not straightforward. As demonstrated in a recent preclinical study, MEK/ERK inhibition in this context results in the increased activation of AKT-ERK5 and therefore does not slow neuroblastoma growth^[56]^. The complexity of the signaling, and escape-signaling pathways involved in neuroblastomas treated with targeted inhibitors underlines the need for a personalized medicine approach to treat these patients.

In anticipation of resistance to ALK inhibition and in order to improve the best response for patients with *ALK* mutant neuroblastoma, combination clinical studies are already underway. The NEPENTHE (Next Generation Personalized Neuroblastoma Therapy, NCT02780128) study is designed to match genomic aberrations found at neuroblastoma relapse with the optimal combination of small molecule targeted treatment. *ALK*-positive patients enrolled onto this study will receive a combination of Ceritinib with the CDK 4/6 inhibitor, Ribociclib. This combination demonstrated strong evidence of synergy in preclinical work, using both *in vitro* experiments to study the effect of the combination on the relevant cyclin D/CDK4/CDK6/RB and ALK signaling pathways, and *in vivo* trials with conventional and patient-derived xenograft models^[57]^. The precise mechanism of interaction between these two compounds is not fully characterized, except that when dosed together in *ALK* mutant cell lines, there was enhanced depletion of both phosphorylated ALK and phosphorylated Rb, compared to either agent alone.

## The relationship between *ALK* and *MYCN* expression and its influence on the design of therapeutic approaches

The mechanism underpinning the relationship between ALK and MYCN is being gradually further characterized, and it is known that ALK stabilizes MYCN protein via the PI3K-Akt pathway^[34,58]^. This strengthens the rationale for the current clinical study of Crizotinib combined with Temsirolimus for patients with *ALK* mutant neuroblastoma. One of the primary objectives of this study is to determine the Phase 2 dose of Crizotinib and Temsirolimus in the setting of relapsed or refractory neuroblastoma with mutations of either *ALK* or MET, as the tyrosine kinase MET is an additional target of Crizotinib. In addition to this the activity of Crizotinib will be recorded alongside pharmacodynamic analysis of biomarkers for PI3K/AKT/mTOR in platelet rich plasma samples and paired tumor samples collected through the course of treatment (ITCC053).

Both wild type ALK and ALK mutant species are able to stimulate the transcription of *MYCN* in neuroblastoma and neuronal cell lines^[59]^. Most recently the transcriptional mediator between ALK and *MYCN* has been identified as “HMG-box transcription factor 1” (HBP1)^[60]^. HBP1 was previously identified as a negative regulator of MYCN activity^[61]^, and also as a component of the *ALK-77* gene signature described by Lambertz *et al.*^[45]^. Further to this, it is now determined that mutant ALK negatively regulates HBP1 through the PI3K-Akt-Foxo3a signaling axis, allowing specific discussion of further targeted combination therapies which should be investigated with a view to disrupting this pathway to the benefit of these ultra-high-risk neuroblastoma patients. To this end, preclinical evidence of synergy has already been presented for several combinations, including the PI3K inhibitor NVP-BEZ235 with the BET-Bromodomain inhibitor, JQ1, allowing for upregulation of HBP1 and suppression of *MYCN* transcription simultaneously.

Whilst it is generally acknowledged that combination therapy will be required to see durable clinical responses, the difficulties associated with moving preclinical combinations to the clinic have led researchers to investigate the potential of dual inhibitors for high-risk patient populations. In particular, the Polo-like kinase 1 (PLK-1) inhibitor BI-2536, which already had low-nanomolar IC_50_ activity against BRD4, has been re-designed to have additional activity against mutant ALK F1174L^[62]^. Through a series of chemical modifications to the structure, an initial set of dual inhibitors has been created, which retain their activity against BRD4, reduce their specificity for PLK-1 and increase their activity and selectivity for the mutant ALK ATP-binding pocket. Rationalizing further these compounds within the *in vivo* preclinical setting, using a panel of validated neuroblastoma models, will provide a compelling case for these compounds to progress into clinical studies.

## Conclusion

Finding a targeted therapy for MYC-activated tumors, including neuroblastoma, has provided an insurmountable challenge for cancer researchers for many years. However, as more is understood about the transcriptional control of MYC expression, within the context of our greater appreciation of the global control of gene expression, it is now highly likely that selective compounds will make their way from bench to bedside. The opportunity to manipulate ALK activity in neuroblastomas is complicated by the specificity of individual ALK mutants for available compounds. However, the progression towards innovative trial designs incorporating personalized genomic medicine and pharmacodynamic markers will undoubtedly improve the translational outcomes.

## Declarations

### Copyright

© The Author(s) 2019.

## References

[1] Morgenstern DA, Potschger U, Moreno L, Papadakis V, Owens C (2018). Risk stratification of high-risk metastatic neuroblastoma: a report from the HR-NBL-1/SIOPEN study.. Pediatr Blood Cancer.

[2] Bresler SC, Weiser DA, Huwe PJ, Park JH, Krytska K (2014). ALK mutations confer differential oncogenic activation and sensitivity to ALK inhibition therapy in neuroblastoma.. Cancer Cell.

[3] De Brouwer S, De Preter K, Kumps C, Zabrocki P, Porcu M (2010). Meta-analysis of neuroblastomas reveals a skewed ALK mutation spectrum in tumors with MYCN amplification.. Clin Cancer Res.

[4] Basta NO, Halliday GC, Makin G, Birch J, Feltbower R (2016). Factors associated with recurrence and survival length following relapse in patients with neuroblastoma.. Br J Cancer.

[5] Barone G, Anderson J, Pearson AD, Petrie K, Chesler L (2013). New strategies in neuroblastoma: therapeutic targeting of MYCN and ALK.. Clin Cancer Res.

[6] Chesler L, Schlieve C, Goldenberg DD, Kenney A, Kim G (2006). Inhibition of phosphatidylinositol 3-kinase destabilizes Mycn protein and blocks malignant progression in neuroblastoma.. Cancer Res.

[7] Vaughan L, Clarke PA, Barker K, Chanthery Y, Gustafson CW (2016). Inhibition of mTOR-kinase destabilizes MYCN and is a potential therapy for MYCN-dependent tumors.. Oncotarget.

[8] Smith JR, Moreno L, Heaton SP, Chesler L, Pearson AD (2016). Novel pharmacodynamic biomarkers for MYCN protein and PI3K/AKT/mTOR pathway signaling in children with neuroblastoma.. Mol Oncol.

[9] Buchel G, Carstensen A, Mak KY, Roeschert I, Leen E (2017). Association with aurora-A controls N-MYC-dependent promoter escape and pause release of RNA polymerase II during the cell cycle.. Cell Rep.

[10] Brockmann M, Poon E, Berry T, Carstensen A, Deubzer HE (2013). Small molecule inhibitors of aurora-a induce proteasomal degradation of N-myc in childhood neuroblastoma.. Cancer Cell.

[11] Gustafson WC, Meyerowitz JC, Nekritz EA, Chen J, Benes C (2014). Drugging MYCN through an allosteric transition in Aurora kinase A.. Cancer Cell.

[12] Otto T, Horn S, Brockmann M, Eilers U, Schuttrumpf L (2009). Stabilization of N-Myc is a critical function of Aurora A in human neuroblastoma.. Cancer Cell.

[13] DuBois SG, Marachelian A, Fox E, Kudgus RA, Reid JM (2016). Phase I study of the aurora A kinase inhibitor alisertib in combination with irinotecan and temozolomide for patients with relapsed or refractory neuroblastoma: a NANT (new approaches to neuroblastoma therapy) trial.. J Clin Oncol.

[14] DuBois SG, Mosse YP, Fox E, Kudgus RA, Reid JM (2018). Phase II trial of alisertib in combination with irinotecan and temozolomide for patients with relapsed or refractory neuroblastoma.. Clin Cancer Res.

[15] Moreno L, Caron H, Geoerger B, Eggert A, Schleiermacher G (2017). Accelerating drug development for neuroblastoma - new drug development strategy: an innovative therapies for children with cancer, European network for cancer research in children and adolescents and international society of paediatric oncology Europe neuroblastoma project.. Expert Opin Drug Discov.

[16] Mosse YP, Fox E, Teachey DT, Reid JM, Safgren SL (2019). A phase II study of alisertib in children with recurrent/refractory solid tumors or leukemia: children’s oncology group phase I and pilot consortium (ADVL0921).. Clin Cancer Res.

[17] Moreno L, Marshall LV, Pearson AD, Morland B, Elliott M (2015). A phase I trial of AT9283 (a selective inhibitor of aurora kinases) in children and adolescents with solid tumors: a Cancer Research UK study.. Clin Cancer Res.

[18] Durbin AD, Zimmerman MW, Dharia NV, Abraham BJ, Iniguez AB (2018). Selective gene dependencies in MYCN-amplified neuroblastoma include the core transcriptional regulatory circuitry.. Nat Genet.

[19] Puissant A, Frumm SM, Alexe G, Bassil CF, Qi J (2013). Targeting MYCN in neuroblastoma by BET bromodomain inhibition.. Cancer Discov.

[20] Yang Z, He N, Zhou Q (2008). Brd4 recruits P-TEFb to chromosomes at late mitosis to promote G1 gene expression and cell cycle progression.. Mol Cell Biol,.

[21] Loven J, Hoke HA, Lin CY, Lau A, Orlando DA (2013). Selective inhibition of tumor oncogenes by disruption of super-enhancers.. Cell.

[22] Henssen A, Althoff K, Odersky A, Beckers A, Koche R (2016). Targeting MYCN-driven transcription by BET-bromodomain inhibition.. Clin Cancer Res.

[23] Chipumuro E, Marco E, Christensen CL, N. Kwiatkowski N, Zhang T (2014). CDK7 inhibition suppresses super-enhancer-linked oncogenic transcription in MYCN-driven cancer.. Cell.

[24] Lucking U, Scholz A, Lienau P, Siemeister G, Kosemund D (2017). Identification of atuveciclib (BAY 1143572), the first highly selective, clinical PTEFb/CDK9 inhibitor for the treatment of cancer.. ChemMedChem,.

[25] Walton MI, Eve PD, Hayes A, Valenti MR, De Haven Brandon AK (2012). CCT244747 is a novel potent and selective CHK1 inhibitor with oral efficacy alone and in combination with genotoxic anticancer drugs.. Clin Cancer Res.

[26] Dolman ME, Poon E, Ebus ME, den Hartog IJ, van Noesel CJ (2015). Cyclin-dependent kinase inhibitor AT7519 as a potential drug for MYCN-dependent neuroblastoma.. Clin Cancer Res,.

[27] Ham J, Costa C, Sano R, Lochmann TL, Sennott EM (2016). Exploitation of the apoptosis-primed state of MYCN-amplified neuroblastoma to develop a potent and specific targeted therapy combination.. Cancer Cell.

[28] Wang H, Hong B, Li X, Deng K, Li H (2017). JQ1 synergizes with the Bcl-2 inhibitor ABT-263 against MYCN-amplified small cell lung cancer.. Oncotarget.

[29] Mosse YP, Laudenslager M, Longo L, Cole KA, Wood A (2008). Identification of ALK as a major familial neuroblastoma predisposition gene.. Nature.

[30] Janoueix-Lerosey I, Lequin D, Brugieres L, Ribeiro A, de Pontual L (2008). Somatic and germline activating mutations of the ALK kinase receptor in neuroblastoma.. Nature.

[31] Mosse YP, Lim MS, Voss SD, Wilner K, Ruffner K (2013). Safety and activity of crizotinib for paediatric patients with refractory solid tumours or anaplastic large-cell lymphoma: a Children’s Oncology Group phase 1 consortium study.. Lancet Oncol.

[32] Chen Y, Takita J, Choi YL, Kato M, Ohira M (2008). Oncogenic mutations of ALK kinase in neuroblastoma.. Nature.

[33] George RE, Sanda T, Hanna M, Frohling S, Luther W (2008). Activating mutations in ALK provide a therapeutic target in neuroblastoma.. Nature.

[34] Berry T, Luther W, Bhatnagar N, Jamin, Poon E (2012). The ALK(F1174L) mutation potentiates the oncogenic activity of MYCN in neuroblastoma.. Cancer Cell.

[35] Zhu S, Lee JS, Guo F, Shin J, Perez-Atayde AR (2012). Activated ALK collaborates with MYCN in neuroblastoma pathogenesis.. Cancer Cell.

[36] Umapathy G, El Wakil A, Witek B, Chesler L, Danielson L (2014). The kinase ALK stimulates the kinase ERK5 to promote the expression of the oncogene MYCN in neuroblastoma.. Sci Signal.

[37] Tucker ER, Tall JR, Danielson LS, Gowan S, Jamin Y (2017). Immunoassays for the quantification of ALK and phosphorylated ALK support the evaluation of on-target ALK inhibitors in neuroblastoma.. Mol Oncol.

[38] Guan J, Fransson S, Siaw JT, Treis D, Van den Eynden J (2018). Clinical response of the novel activating ALK-I1171T mutation in neuroblastoma to the ALK inhibitor ceritinib.. Cold Spring Harb Mol Case Stud.

[39] Bresler SC, Wood AC, Haglund EA, Courtright J, Belcastro LT (2011). Differential inhibitor sensitivity of anaplastic lymphoma kinase variants found in neuroblastoma.. Sci Transl Med,.

[40] Guan J, Tucker ER, Wan H, Chand D, Danielson LS (2016). The ALK inhibitor PF-06463922 is effective as a single agent in neuroblastoma driven by expression of ALK and MYCN.. Dis Model Mech.

[41] Infarinato NR, Park JH, Krytska K, Ryles HT, Sano R (2016). The ALK/ROS1 inhibitor PF-06463922 overcomes primary resistance to crizotinib in ALK-driven neuroblastoma.. Cancer Discov.

[42] Johnson TW, Richardson PF, Bailey S, Brooun A, Burke BJ (2014). Discovery of (10R)-7-amino-12-fluoro-2,10,16-trimethyl-15-oxo-10,15,16,17-tetrahydro-2H-8,4-(m etheno)pyrazolo[4,3-h][2,5,11]-benzoxadiazacyclotetradecine-3-carbonitrile (PF-06463922), a macrocyclic inhibitor of anaplastic lymphoma kinase (ALK) and c-ros oncogene 1 (ROS1) with preclinical brain exposure and broad-spectrum potency against ALK-resistant mutations.. J Med Chem.

[43] Heath JA, Campbel MA, Thomas A, Solomon B (2018). Good clinical response to alectinib, a second generation ALK inhibitor, in refractory neuroblastoma.. Pediatr Blood Cancer.

[44] Pacenta HL, Macy ME (2018). Entrectinib and other ALK/TRK inhibitors for the treatment of neuroblastoma.. Drug Des Devel Ther.

[45] Lambertz I, Kumps C, Claeys S, Lindner S, Beckers A (2015). Upregulation of MAPK negative feedback regulators and RET in mutant ALK neuroblastoma: implications for targeted treatment.. Clin Cancer Res.

[46] Krytska K, Ryles HT, Sano R, Raman P, Infarinato NR (2016). Crizotinib Synergizes with chemotherapy in preclinical models of neuroblastoma.. Clin Cancer Res.

[47] Carr-Wilkinson J, O’Toole K, Wood KM, Challen CC, Baker AG (2010). High frequency of p53/MDM2/p14ARF pathway abnormalities in relapsed neuroblastoma.. Clin Cancer Res.

[48] Yoda S, Lin JJ, Lawrence MS, Burke BJ, Friboulet L (2018). Sequential ALK inhibitors can select for lorlatinib-resistant compound ALK mutations in ALK-positive lung cancer.. Cancer Discov.

[49] Shaw AT, Friboulet L, Leshchiner I, Gainor JF, Bergqvist S (2016). Resensitization to crizotinib by the lorlatinib ALK resistance mutation L1198F.. N Engl J Med.

[50] Debruyne DN, Bhatnagar N, Sharma B, Luther W, Moore NF (2016). ALK inhibitor resistance in ALK(F1174L)-driven neuroblastoma is associated with AXL activation and induction of EMT.. Oncogene.

[51] Redaelli S, Ceccon M, Zappa M, Sharma GG, Mastini C (2018). Lorlatinib treatment elicits multiple on- and off-target mechanisms of resistance in ALK-driven cancer.. Cancer Res.

[52] Padovan-Merhar OM, Raman P, Ostrovnaya I, Kalletla K, Rubnitz KR (2016). Enrichment of targetable mutations in the relapsed neuroblastoma genome.. PLoS Genet.

[53] Chen L, Humphreys A, Turnbull L, Bellini A, Schleiermacher G (2016). Identification of different ALK mutations in a pair of neuroblastoma cell lines established at diagnosis and relapse.. Oncotarget.

[54] Eleveld TF, Oldridge DA, Bernard V, Koster J, Colmet Daage L (2015). Relapsed neuroblastomas show frequent RAS-MAPK pathway mutations.. Nat Genet.

[55] Schleiermacher G, Javanmardi N, Bernard V, Leroy Q, Cappo J (2014). Emergence of new ALK mutations at relapse of neuroblastoma.. J Clin Oncol.

[56] Umapathy G, Guan J, Gustafsson DE, Javanmardi N, Cervantes-Madrid D (2017). MEK inhibitor trametinib does not prevent the growth of anaplastic lymphoma kinase (ALK)-addicted neuroblastomas.. Sci Signal,.

[57] Wood AC, Krytska K, Ryles HT, Infarinato NR, Sano R (2017). Dual ALK and CDK4/6 inhibition demonstrates synergy against neuroblastoma.. Clin Cancer Res.

[58] Moore NF, Azarova AM, Bhatnagar N, Ross KN, Drake LE (2014). Molecular rationale for the use of PI3K/AKT/mTOR pathway inhibitors in combination with crizotinib in ALK-mutated neuroblastoma.. Oncotarget.

[59] Schonherr C, Ruuth K, Kamaraj S, Wang CL, Yang HL (2012). Anaplastic lymphoma kinase (ALK) regulates initiation of transcription of MYCN in neuroblastoma cells.. Oncogene.

[60] Claeys S, Denecker G, Durinck K, Decaesteker B, Mus LM (2019). ALK positively regulates MYCN activity through repression of HBP1 expression.. Oncogene.

[61] Escamilla-Powers JR, Daniel CJ, Farrell A, Taylor K, Zhang X (2010). The tumor suppressor protein HBP1 is a novel c-myc-binding protein that negatively regulates c-myc transcriptional activity.. J Biol Chem.

[62] Watts E, Heidenreich D, Tucker E, Raab M, Strebhardt K (2019). Designing dual inhibitors of anaplastic lymphoma kinase (ALK) and bromodomain-4 (BRD4) by tuning kinase selectivity.. J Med Chem.

